# Term Identification Methods for Consumer Health Vocabulary Development

**DOI:** 10.2196/jmir.9.1.e4

**Published:** 2007-03-14

**Authors:** Qing T Zeng, Tony Tse, Guy Divita, Alla Keselman, Jon Crowell, Allen C Browne, Sergey Goryachev, Long Ngo

**Affiliations:** ^4^AquilentIncLaurelMDUSA; ^3^Management Systems DesignersIncFairfaxVAUSA; ^2^LHNCBCNational Library of MedicineNIHDHHSBethesdaMDUSA; ^1^Decision Systems GroupBrigham and Women’s HospitalHarvard Medical SchoolBostonMAUSA

**Keywords:** Consumer health information, vocabulary, natural language processing

## Abstract

**Background:**

The development of consumer health information applications such as health education websites has motivated the research on consumer health vocabulary (CHV). Term identification is a critical task in vocabulary development. Because of the heterogeneity and ambiguity of consumer expressions, term identification for CHV is more challenging than for professional health vocabularies.

**Objective:**

For the development of a CHV, we explored several term identification methods, including collaborative human review and automated term recognition methods.

**Methods:**

A set of criteria was established to ensure consistency in the collaborative review, which analyzed 1893 strings. Using the results from the human review, we tested two automated methods—C-value formula and a logistic regression model.

**Results:**

The study identified 753 consumer terms and found the logistic regression model to be highly effective for CHV term identification (area under the receiver operating characteristic curve = 95.5%).

**Conclusions:**

The collaborative human review and logistic regression methods were effective for identifying terms for CHV development.

## Introduction

Two important steps in vocabulary development are (1) the identification of candidate strings (ie, words or phrases) in a domain and (2) the determination of which of these should be included in a vocabulary as “valid” terms, also called “termhood determination.” Health vocabulary development, which has a long history, requires significant effort for collecting candidate terms and determining termhood [1]. While vocabularies such as SNOMED (Systematized Nomenclature of Medicine) and ICD-9 (International Classification of Diseases, Ninth Revision) include many health terms, there is no consensus on termhood criteria (ie, what constitutes a “term”) [2]. The decision to include terms in a vocabulary is made for a particular domain for certain tasks (eg, indexing or billing). Thus, the review criteria and procedures used by vocabulary developers, which are often not published, inevitably differ. Terms included in health vocabularies also vary significantly. For instance, in the Unified Medical Language System (UMLS), the same concept is often represented in various source vocabularies by different terms. The terms “head ache” and “cranial pain” are both synonyms of the UMLS concept “headache.” The source vocabulary for “head ache” is DXplain, and the source vocabulary for “cranial pain” is MeSH (medical subject heading).

Research and development of controlled consumer health vocabularies (CHVs) is a relatively new endeavor in the health vocabulary field [3]. In the general biomedical literature, research on consumer understanding of medical words and concepts has focused primarily on relatively short lists of discrete terms in various specialties. In the informatics domain, a few companies (eg, Apelon and WellMed) offer proprietary CHV products, though these products have not been publicly evaluated.

The general goal of our CHV research is to help overcome the vocabulary gap between consumers and health information provided by informatics applications. The specific aim of this paper is to elucidate term identification methods for CHVs. CHV research has largely been driven by the proliferation of health-related materials on the Web, the emergence of electronic personal health records, as well as the growing availability of various consumer health applications (eg, decision support tools). Over the past five years, researchers have found that consumer terms are not well covered by the existing health vocabularies, which mostly represent the language of health professionals [4-9]. Indeed, expressions used by consumers to describe health-related concepts and relationships among such concepts frequently differ on multiple levels (ie, syntactic, conceptual, and explanatory) from those of professionals. Thus, consumer health informatics research and application development will benefit from the development of CHVs.

Developing and validating a comprehensive CHV is challenging because “consumers” constitute a plethora of highly diverse groups. Further, individuals uniquely acquire health-related terms and concepts from formal and informal sources (eg, media exposure) and from personal experiences. Nevertheless, there is strong evidence of the stability of lay health language among particular populations, for specific tasks [3].

We have been working on an open access and collaborative (OAC) CHV project. The first step in creating the OAC CHV was to identify consumer terms since surface forms, represented as strings in written text, are more tractable than concepts (ie, underlying meanings) or semantic relations, both of which require in-depth understanding of term usage, rhetorical intent, and explanatory models. Because consumer terms are heterogeneous and even less well defined than professional terms [10], the termhood determination task proved to be particularly challenging. Our term identification effort has been guided by two principles:

1. CHVs consist of actual terms commonly used by consumers (in any particular discourse group).

2. CHV terms must allow for computer processing of consumer language.

Since many professional health vocabulary terms are already used by consumers, though in some cases with different or broader semantics (eg, “diabetes” for diabetes mellitus, types 1 and 2), we focused on consumer terms not yet represented in existing vocabularies (eg, “broken finger” for any type of fracture in the “distal,” “middle,” or “proximal phalanges”).

Because the number of candidate strings is often very large in any domain, researchers have explored the use of corpus-based automated term recognition (ATR) methods for extracting the most promising strings for human review from domain-specific documents [11, 12]. ATRs vary from statistical or information theory–based approaches (eg, *t* test) [13] to syntax-based methods (eg, noun phrase extraction and context analysis) [14] and hybrid mechanisms (eg, C-value formula) [15, 16]. Both the *t* test and the C-value formula have been used successfully in termhood determination. Such studies reinforce the general notion that strings typically considered as terms share some common characteristics, such as words in a term tend to occur more frequently together, terms are often noun phrases, and terms may be part of several longer strings.

In the biomedical domain, ATR methods have been applied to Medline literature [17] and clinical reports [15]. While most ATR methods outside the biomedical domain were designed to be general purpose, biomedical ATR methods tend to be more narrowly focused [18]. The type of terms targeted by ATR vary, including gene and protein names in a number of recent studies [18-21].

In this study, we first identified CHV terms through collaborative review of strings derived from query logs of a consumer health site [22]. Because of the considerable variability in lay health expressions, standardized review criteria and procedures to ensure consistency in selecting CHV terms were developed. After obtaining the human-reviewed n-grams (ie, n word strings), we experimented with two ATR methods: logistic regression and the C-value formula. The initial features used in the regression model were informed by existing ATR methods, in particular, the C-value model [16] and the termhood formula proposed by Wermter and Hahn [12]. We also evaluated the popular C-value method.

Our use of ATRs in this study differs from that in prior studies in the biomedical domain in two aspects: (1) short phrases from query logs were used as the text corpus rather than entire sentences from full-text sources, and (2) “new” CHV terms, not yet part of existing vocabularies, were identified rather than “pre-existing” terms such as UMLS terms.

## Methods

The term identification study had three components:

1. Candidate string extraction from a query log data set of terms that could not be mapped to UMLS
2. Collaborative manual review of a subset of the candidate strings and identification of CHV terms
3. Application of ATR methods (the C-value formula and logistic regression models) to human-reviewed CHV terms

### Candidate String Extraction

We obtained a set of query log files [22] from the MedlinePlus site covering the period from October 2002 to October 2003, courtesy of the National Library of Medicine (NLM). The log data were preprocessed to filter out all queries that were not in English, appeared to be machine generated (eg, very large numbers of queries from the same IP address within a minute), and that were redundant (ie, from the same host at time intervals of less than 5 minutes).

The preprocessed queries were then mapped to the 2004AA version of the UMLS Metathesaurus using lexical methods (ie, removing non-alphanumeric symbols, stemming, normalization, and truncation). Queries that did not map to the UMLS Metathesaurus were broken into n-grams. N-grams that matched terms in the Metathesaurus were removed, and the remaining n-grams were collected into sets by frequency and number of words.

We used n-gram analysis to find candidate terms from unmapped query strings. The n-gram analysis uses the frequencies of n-grams and text fragments of n words in a text sample to estimate the likelihood that a string is a potential term. In general, the more frequently an n-gram appears in text documents, the increased likelihood that the n-gram is a “useful” term.

### Collaborative Manual Review

**Figure 1:**
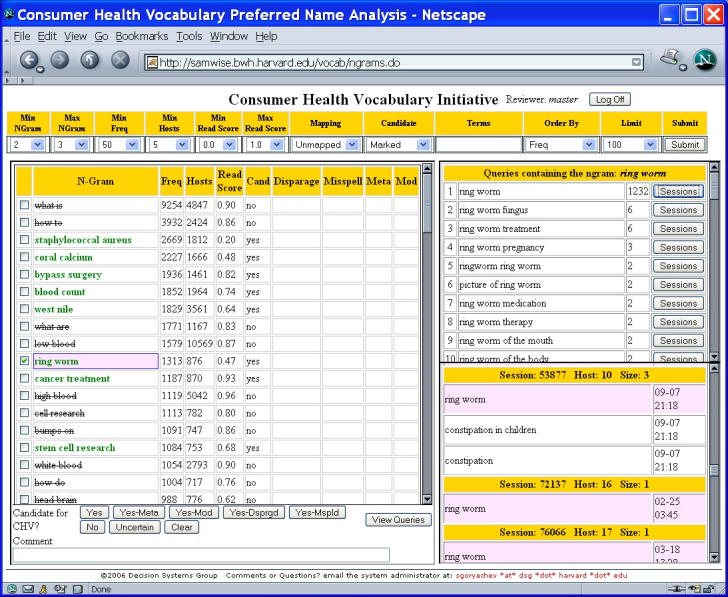
Application to support collaborative manual review of candidate strings

Six researchers (first six of the authors) reviewed candidate strings (n-grams) collaboratively. First, each reviewer independently reviewed a subset of the n-grams (n = 1 to 4 and frequency > 50) and voted on whether they should be considered CHV terms. Unanimous votes for n-grams that were reviewed by at least three people were entered as “master” votes. Otherwise, termhood was discussed by the entire group until consensus was reached and a master vote was cast. To support reviewers from geographically distributed locations and to calculate votes, a specially designed Web-based application [23] was utilized (Figure 1).

Through several iterations of votes and discussion, we established the following review criteria:

1. CHV terms should be syntactic constituents or phrases such as a noun phrase or adjective phrase (eg, “bypass surgery” is a phrase, but “fever in” is not). Special attention should be given to noun phrases.
2. CHV terms should have independent semantics and should not only occur as a part of longer valid terms or as a part of wild card searches (eg, [chicken-, small-] “pox vaccine” is not considered a CHV term).
3. CHV terms should be specific to the medical domain (eg, “Google” and “Yahoo” are general words, not CHV terms).
4. CHV terms should function as semantic components in addition to functioning as syntactic components (eg, stop words “the” and “a” as well as empty verbs “make” and “take” are not considered CHV terms).
5. N-grams representing existing UMLS medical concepts are considered to be CHV terms, but CHV terms may represent non-UMLS concepts.
6. Eponymous forms of CHV terms are considered to be CHV terms (eg, “Parkinson’s”).
7. CHV terms may include spelling errors, (eg, “Chron's disease”). These misspelled terms are given the label “disparaged.”
8. Terms with distinct clinical semantics (eg, “result”) are considered to be CHV terms, regardless of ambiguity and/or vagueness in other domains.

We singled out several types of terms for future investigation and assigned special labels to them:

- meta: A term that is usually used to indicate the category/type of information sought or presented (eg, “picture,” “guideline,” and “tutorial”).
- modifier: A term not typically used by itself, but for limiting or qualifying other terms (eg, “sexually” as in “sexually active”).
- relation: A term not typically used by itself, but used to describe relations among concepts (eg, “caused by” and “results in”). We also include the unary relation “not” in this set.

Currently, we consider terms classified as meta and modifier to be CHV terms, but relations are not considered CHV terms.

Once these review criteria were established, researchers double-checked the previously cast master votes for compliance. A second round of discussion resulted in some adjustments to the votes.

### Application of Automated Term Recognition (ATR)

We explored the use of two ATR methods to facilitate candidate selection for human review: (1) the C-value method (C loosely stands for “candidate collection”) and (2) logistic regression.

We applied the C-value method to the strings that had already been reviewed. First, the strings were parsed to filter out single-word strings and strings that were not noun phrases. The C-value was calculated using the formula [16] given in Textbox 1.

**The C-value was calculated using this formula**

*C-value(**a**) =* log_­­­2_|**a**|*f(**a**) if ***a*** is not nested

(When ***a*** is a substring of ***b,*** we refer to ***a*** as nested and ***b*** as ***a***’s nesting string.)

*C-value(**a**) =* log­­­_2_|**a**|*(f(**a**) – 1/p(T**a**)*sum*(f(**b**)))* if ***a*** is nested

***a** =* candidate string (eg, “failure”)

***b** =* nesting strings (eg, “heart failure”)

*|**a**| =* length (number of words) of ***a***

*f(**a**) =* frequency of ***a*** in the corpus

*T**a** =* set of ***b*** that contain ***a***

*P(T**a**) =* number of ***b*** in *T**a***

*f(**b**) =* frequency of ***b*** in the corpus

To create the logistic regression model that predicts the termhood of a candidate string ***a,*** we explored syntactic category, frequency of occurrence, string length, word count and number, frequency and termhood status of ***a***’s nesting, and nested strings as variables and used the master vote as outcome. Human-reviewed strings were used as the training and testing data sets. The initial feature variables were as follows:

1. part-of-speech (POS) tag (eg, noun or adjective) of the first word
2. POS tag of the last word
3. noun phrase status (ie, yes/no)
4. word count (ie, number of words in ***a*)**
5. number of distinct ***a***’s nesting string ***b***
6. number of repeated ***b***
7. percentage of distinct ***b*** that are known valid (UMLS) terms
8. percentage of repeated ***b*** that are known valid (UMLS) terms
9. number of distinct ***a***’s nested string ***c***
10. number of repeated ***c***
11. percentage of distinct ***c*** that are known valid (UMLS) terms
12. percentage of repeated ***c*** that are known valid (UMLS) terms
13. frequency of ***a***
14. number of distinct host ***h*** that ***a*** originated from
15. average number of distinct queries containing ***a*** per host

The frequency distribution of the POS tags (variables 1 and 2) required them to be collapsed into fewer categories for modeling. The original tags came from a Brill-style, rule-based POS tagger developed by Mark Hepple [24]. We first transformed them into a smaller set of tags used by the UMLS SPECIALIST Lexicon of the National Library of Medicine (NLM) [25]. (Details of the transformation rules can be found in [26].) Several tags appeared with low frequency and were then merged: the tags AUXILARY and MODAL were merged with VERB, and the tags CONJUNCTION, DETERMINER, NUMBER, SYM, UNKNOWN, PRONOUN, and PREP were merged into a new category, OTHER.

The continuous variables (variables 4 to 15) were dichotomized based on the median value. The dichotomized variables were used in the logistic regression to predict or explain the probability of having a term voted “yes” for termhood.

The logistic regression model building was carried out by a stepwise procedure. After calculating the odds ratio estimates, most of the variables were dropped. The remaining variables 1, 2, 3, 6, 10, and 15 were represented in the regression formula as FirstPOS, LastPOS, np_value, repeat_sup_gt_median, repeat_sub_gt_median, and distinct_perhost_gt_median.

For both the C-value formula and the regression model, we calculated the sensitivity and specificity at different thresholds to create the receiver operating characteristic (ROC) curves. To estimate the area under the ROC curve for the logistic regression, we used the c-statistic [27] (note that this is not the same as C-value). It has the following meaning. From the final multivariable logistic regression model, the predicted probability of the termhood voted “yes” can be computed for each term. For any two terms, one with vote “yes” and one with vote “no,” if the predicted probability for vote “yes” is higher than the predicted probability for vote “no,” then we have a concordant pair. If the predicted probability of vote “no” is higher, then we have a discordant pair. If the pair is neither concordant nor discordant, then it is tied. Let *T* be the total number of all possible yes-no pairs of all terms. Let *C* be the number of concordant pairs, and *D* the number of discordant pairs. The c-statistic is calculated as *c* = (*C* + 0.5(*T* − *C* − *D*)) / *T*.

## Results

We identified 18454 candidate n-grams (n = 1 to 5); 7967 were reviewed by at least one reviewer, and 1893 distinct n-grams received master votes (Table 1). Among the n-grams with master votes, 23 were meta, 39 were modifier, and 5 were relation.

**Table 1:** Number of n-grams with master votes and number of n-grams voted as CHV terms

**N-gram**	**Number of Master Votes**	**Number of CHV Terms**
1-gram	379	261
2-gram	1101	303
3-gram	356	154
4-gram	57	35
**Total**	**1893**	**753**

**Figure 2:**
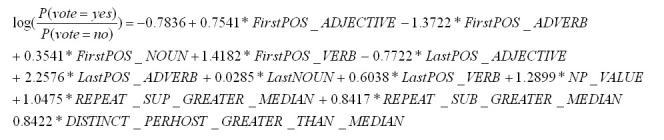
The logistic regression model

 The logistic regression model is shown in Figure 2. In this logistic regression model, syntactic information (first 9 variables) and nesting pattern (last 3 variables) determine the termhood. The importance of syntactic information has long been recognized by models like the C-value. Conspicuously, word count and frequency are missing from our model, though longer and more frequent strings are more likely to be considered terms. To a large extent, length and frequency are reflected by the nesting patterns: very short strings are likely to be part of many nesting strings, and less frequent strings are likely to be coincidental combinations of more common words, meaning that it would have more nested strings.

**Figure 3:**
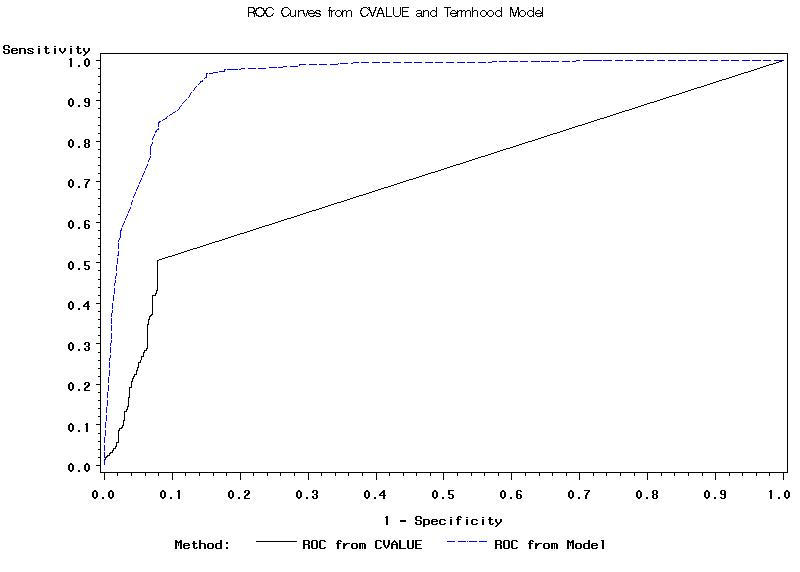
Curves for C-value and the regression model

The ROC curves for C-value and the regression model are shown in Figure 3. The area under the ROC curve (AUC) is 70.9% for the C-value method and 95.5% for the regression model. Higher AUC signifies increased distinguishing power: 100% = perfect discriminative ability, 50% = no ability, < 50% = predications were made in the wrong direction. Thus, the AUC results suggest the regression model to be very effective and better than the C-value for identifying CHV terms.

## Discussion

 This paper reports on several term identification methods for the OAC CHV project. We established a set of criteria and procedures to conduct a manual review, resulting in multiple reviewers reaching consensus on 1893 n-grams, including identification of 753 new terms for inclusion in the OAC CHV that were not in the 2004AC version of UMLS.

The OAC termhood criteria were established collaboratively, reflecting the reviewers’ backgrounds in several different fields: controlled vocabulary, health informatics, linguistics, cognitive science, and computer science. While the OAC termhood criteria could be further refined and termhood criteria for health vocabularies are often not published, we believe publishing such criteria could benefit vocabulary research. For instance, many articles evaluate vocabularies and study methods of mapping one vocabulary to another [28-31]. These evaluations and mapping methods could be better guided by the termhood criteria of target vocabularies.

In CHV research, the termhood issue is of particular importance because there has been limited discussion and little consensus on what should be considered a consumer term. Is “sun poisoning” an acceptable term? How about “skin conditions?” As was pointed out in the Introduction, health professional vocabularies do not always agree on the termhood of a phrase. Consumer expressions, however, require more scrutiny because it is harder to determine their semantics and contexts of usage.

We tested two ATR methods (C-value and logistic regression) on the human-reviewed n-grams. The C-value was useful for determining termhood, though it did not have high distinguishing power (AUC = 70.9%). The AUC for the logistic regression model was 95.5%, which is fairly satisfactory.

These results suggest that a specially fitted logistic regression model is better suited than the generic C-value method for the task of identifying CHV terms according to our criteria. The C-value method’s performance problem was partially caused by issues unique to this data set, among them the inclusion of infrequent misspellings and the high frequency of most candidates, which made frequency a less reliable predicator. The imperfection in noun-phrase parsing is not unexpected, though the relatively short query string posed a greater challenge for parsing. Like many vocabularies, OAC includes strings that are single words and are not noun phrases, while C-value is typically calculated for multiword noun phrases.

The logistic regression model demonstrated excellent suitability for OAC termhood determination. It may have to be altered to be used with other corpora or for other types of vocabularies due to the particularities of query-based corpus attributes such as the short length of the documents. Nonetheless, training of predictive models for a particular corpus and vocabulary is a generalizable strategy. Although general principles exist, the determination of which strings are to be considered legitimate vocabulary terms often depends on the domain and the vocabulary developers’ criteria (eg, including verb phrases [15] or not).

The regression model utilizes syntactic and nesting pattern features; both types of features are well-recognized termhood indicators. A concern often raised about CHV research is that the syntax and semantic of consumer phrases are too unruly to be represented in a computable vocabulary. The fact that many consumer phrases have common term characteristics suggests that they are tractable terms.

Our study has several limitations. Because consumer utterances are not readily available as corpora of medical literature or clinical records, we used query logs that contained relatively few complete sentences. Subsequently, this resulted in many POS and noun phrase analysis errors. As well, we only had researchers and not lay consumers review the candidate terms, due to budget and logistic constraints. However, the analysis was based on utterances from queries submitted by tens of thousands of consumers.

Based on the result of this study, we plan to apply the logistic regression model to the candidate n-grams and select those predicted to be terms for human review. We also plan to add the identified CHV terms to OAC. The authors associated with NLM are interested in investigating similar techniques to aid in identifying candidate terms for inclusion into the SPECIALIST Lexicon of the NLM, and for quality control.

## Abbreviations

ATR: automated text recognition
AUC: area under the curve
CHV: consumer health vocabulary
NIH: National Institutes of Health
NLM: National Library of Medicine
OAC: open access and collaborative
POS: part of speech
ROC: receiver operating characteristic
UMLS: Unified Medical Language System
